# The sources and types of innovation knowledge in technology adoption decisions in infection prevention and control – comparative case studies of 12 NHS trusts in England

**DOI:** 10.1186/1753-6561-5-S6-O47

**Published:** 2011-06-29

**Authors:** Y Kyratsis, R Ahmad, A Holmes

**Affiliations:** 1 Faculty of Medicine, Imperial College London, London, UK

## Introduction / objectives

The nature, sources and format of evidence used by managers and clinicians is important in introducing innovations in healthcare. We investigate the organisational decision making process focusing on the adoption of innovative technologies in the context of infection prevention and control (IPC) and the nature of evidence used.

## Methods

Qualitative, multi-level, multiple case study design involving primary & acute care trusts. We conducted 121 semi-structured interviews drawing on a purposive multi-level, multi-stakeholder sample. Data was analysed using an integrated approach.

## Results

We mapped out 38 organisational technology selection decisions from July 2009 to August 2010. We specifically mapped the organisational adoption decisions to three types of innovation knowledge: 'awareness' (awareness that the innovation exists), 'principles' (its functioning principles) and 'how to' (information related to its practical use). The leadership role adopted by the Director of IPC and the professional background of key decision makers influenced this asymmetry to different types of knowledge considered.

## Conclusion

In the commercial sector innovation adoption focuses at the individual level and majority of attention by change agencies is around *awareness* and *how to* knowledge. In our study we found the converse; overall less attention was given to *how to* knowledge at the point of innovation adoption decision and more attention was attributed to *principles* knowledge both by decision makers and change agents. Attending to ‘how to’ knowledge at decision making stage may enhance successful technology adoption and effective use. This has important implications for suppliers, managers and clinicians.

## Disclosure of interest

None declared.

